# Effectiveness of Virtual Reality in Reducing Pain and Stress During Office Hysteroscopy: A Randomized Controlled Trial

**DOI:** 10.3390/healthcare13020131

**Published:** 2025-01-12

**Authors:** Josep Estadella Tarriel, Josep Perelló Capó, Marta Simó González, Marta Bailón Queiruga, Jordi Real Gatius, Mar Gomis-Pastor, Diana Marre, Elisa Llurba Olivé

**Affiliations:** 1 Obstetrics & Gynecology Department, Hospital Universitari de la Santa Creu i Sant Pau, 08025 Barcelona, Spain; 2 Pediatrics, Obstetrics and Gynecology, and Preventive Medicine and Public Health Department, Universitat Autònoma de Barcelona, 08193 Barcelona, Spain; 3 Obstetrics & Gynecology Department, Hospital Universitari Arnau de Vilanova, 25198 Lleida, Spain; 4 Obstetrics & Gynecology Department, Hospital Universitari Josep Trueta, 17007 Girona, Spain; 5 Digital Health Validation Center, Hospital de la Santa Creu i Sant Pau, Sant Pau Campus Salut Barcelona, 08041 Barcelona, Spain; 6 Institut de Recerca Sant Pau (IR SANT PAU), Sant Quintí 77 79, 08041 Barcelona, Spain; 7 Department of Social and Cultural Anthropology, Universitat Autònoma de Barcelona, Bellaterra, 08193 Barcelona, Spain

**Keywords:** office hysteroscopy, virtual reality, pain, anxiety, stress, gynecology, analgesia, trial

## Abstract

**Introduction:** Office hysteroscopy (OH) offers a “see and treat” strategy, enabling most gynecological conditions to be addressed outside the operating room without anesthesia. Despite its convenience, the associated pain and stress remain significant barriers to its widespread success among women. Both pharmacological and non-pharmacological interventions have been explored to mitigate these challenges, albeit with mixed outcomes. **Methods:** This study aimed to evaluate the effectiveness of virtual reality (VR) in reducing pain and stress associated with OH using objective measurements. **Results:** Our findings indicate that VR significantly reduced pain during OH compared to the control group (−1.08, 95%CI; −1.93–0.23, on the Visual Analogue Scale (VAS), *p* = 0.013) and 10 min post-procedure (−1.24, 95%CI; −1.99–0.48, *p* = 0.001), without significant effects on stress-related variables. Stratified analyses further revealed that the efficacy of VR in pain reduction is influenced by individual patient characteristics, with greater effectiveness observed in women with lower baseline stress, premenopausal status and a history of childbirth, regardless of vaginal delivery. **Conclusions:** VR represents a promising strategy for managing OH-associated pain, with its effectiveness largely depending on patient-specific variables.

## 1. Introduction

Hysteroscopy is a minimally invasive endoscopic technique that enables direct visualization of the uterine cavity and constitutes the gold standard for the diagnosis and treatment of most intrauterine pathologies, such as endometrial polyps, submucosal fibroids or uterine malformations [1]. Improvements in instrumentation and techniques have enabled these procedures to be performed in an office setting without anesthesia [2]. Office hysteroscopy (OH) marked a significant paradigm shift in gynecological interventions [3], notably reducing the costs associated with traditional operating-room procedures [4] and allowing for a “see and treat” approach, enabling therapeutic intervention immediately after observing any pathological condition in the same session [5]. Additionally, OH without anesthesia provides a quick recovery and a faster reincorporation of the patient into normal daily activities. OH has proven highly effective in addressing most gynecological conditions [1,6] with a 90% success rate [7]. Despite its benefits, the primary limitation of OH remains the pain associated with the procedure, reported in many cases as moderate and severe [8]. This significantly affects patient’s tolerance and occasionally leads to discontinuation of the technique [9,10].

Several factors may affect patients’ tolerance of the OH procedure, such as vaginal delivery history, menopausal status or chronic pain [11,12,13]. In addition to these factors, anxiety plays a crucial role in influencing the perception of pain and, therefore, in the acceptance of the procedure [14,15]. Several studies have shown that longer waiting times before the procedure [16] and the low expertise of the specialist performing OH [9] can increase patient anxiety and pain perception. Therefore, addressing both factors is essential for improving the overall acceptance and success of the procedure and highlights the need for effective strategies applicable to all patients.

Efforts to alleviate pain and anxiety associated with OH have led to the exploration of several pharmacological strategies, including nonsteroidal anti-inflammatory drugs (NSAIDs), local analgesics, opioids and paracervical blocks [17]. However, given the subjective nature of pain perception [17], there is extensive heterogeneity in the results obtained for each therapeutic approach [5,18,19,20,21].

Relatively recent efforts have focused on non-pharmacological strategies for pain reduction. A particularly promising innovation is the use of virtual reality (VR) devices that can recreate three-dimensional (3D) immersive environments. This technology is becoming increasingly accessible in medical settings, demonstrating success in reducing pain and anxiety for patients undergoing treatments like burn wound care, dental procedures, labour, and minor gynecological procedures [22,23,24,25,26,27]. However, when applied to OH, there are few studies published on this topic, with a low number of patients and inconsistent results. Some studies have highlighted its effectiveness in alleviating discomfort [8,28], while others reported no significant improvements in pain and anxiety levels [29,30,31]. The overall conclusion of these studies indicates the necessity for further research involving a larger sample size to enhance the generalizability of the findings.

For this reason, in this study, we aimed to assess the efficacy of VR in OH patients, focusing on pain and stress levels and using objective measures to overcome the limitations of previous research. In the following sections, we will describe the materials and methods used in the study, followed by the presentation of the results. Finally, a comprehensive review and comparison of the relevant studies has been conducted and will be presented in detail in the Section 4, providing the necessary context for the interpretation of the results.

## 2. Materials and Methods

### 2.1. Study Design

**Figure 1:**
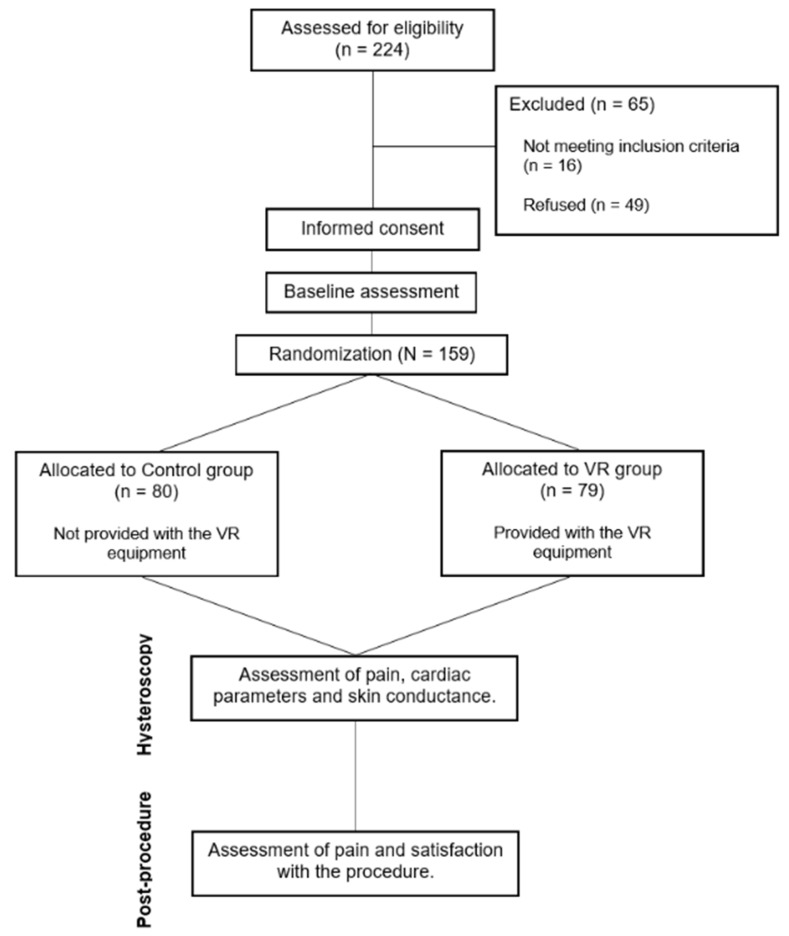
CONSORT diagram of the study.

An unblinded, randomized, parallel-group and open clinical trial was conducted at two tertiary hospitals (Santa Creu i Sant Pau Hospital. Barcelona, Spain; and Arnau de Vilanova Hospital. Lleida, Spain) between February 2020 and June 2023 (Figure 1). The study was approved by and regulated by the ethics committees of both centres and registered on ClinicalTrials (code NCT04721587).

### 2.2. Study Participants and Eligibility Criteria

Patients scheduled for an office hysteroscopy were invited to participate in the study and were selected based on the eligibility criteria: over 18 years of age, able to understand and accept the study procedures (hysteroscopy, 3D VR protocol pre- and during the procedure, physiological measurements of stress- and pain-related parameters, anxiety, pain and satisfaction reports regarding the procedure), and not taking anxiolytic treatment. All participants signed the informed consent and were excluded from the clinical trial if they met one or more of the following exclusion criteria: inability to understand the study’s characteristics and procedures; under 18 years of age; pregnant; and having a prior diagnosis of an anxiety disorder (or being under anxiolytic treatment), psychosis, or other severe mental disorders due to the close relationship between anxiety and the perceived level of pain, as well as the modulation of these levels by anxiolytic drug,; additionally, as recommended by VR device manufacturers, we excluded women suffering from vertigo, epilepsy, or an active ear infection or with a diagnosis of arterial hypertension or the presence of cardiovascular diseases as they should not use this technology due to the potential risk of exacerbating their underlying condition.

### 2.3. Recruitment and Randomization Process

After accepting participation in the study and signing the informed consent, participants were randomly allocated a group through a secure computer online system with a randomization scheme based on a permuted block of random block sizes (Clinapsis software v.1 [32], which was applied to assist in the design and management of epidemiological and clinical studies and designed by the Statistics and Methodological support unit of the Research Institute of Santa Creu i Sant Pau Hospital); this ensured equal probability for both interventions. Due to the nature of the study, blinding of patients or healthcare professionals was not feasible; however, the allocation was concealed until randomization. Those patients in the intervention group were equipped with a portable, standalone VR headset PICO G2 (Pico XR, Mountain View, CA). Women allocated to the control group did not receive the VR headsets, and the OH was performed as a routine procedure. The required sample size for this study was determined based on a minimum detectable difference of 1.5 points on the VAS for pain perception, with an assumed standard deviation (SD) of 3 points. A potential rate of non-assessable cases below 10% was considered, alongside a probability of a type I error set at the usual value of 5% (alpha of 0.05), with a minimum required power of 80% (type II error, beta of 0.20). The sample size was calculated as 160 participants; however, due to recruitment constraints related to the COVID-19 pandemic, the final enrollment included 80 patients in the CTL group and 79 in the VR group.

### 2.4. Hysteroscopy Procedure

All procedures were performed in an office setting according to the centres’ standard clinical practice. The procedures were performed by four experienced consultant gynecologists (JE, MB, MS, JP). Hysteroscopic instrumentation (scissors and graspers, tissue removal devices, bioplar electrodes) was selected by the facultative based on patient’s pathology and the specific procedure required, following clinical criteria. The different hysteroscopes available were 5.0 mm (Truclear 5C) or 4.3 mm (Bettochi) rigid hysteroscopes. Hysteroscopy was performed using a vaginoscopic approach (without speculum or cervical tenaculum), with 0.9% saline solution used as a distension medium with pressures ranging 80–100 mmHg. Thirty minutes before the hysteroscopy, all the participants were administered a single 600 mg dose of Ibuprofen and a single 2.5 mg dose of Diazepam, as per routine clinical practice. No additional local anesthesia or recovery analgesia was administered beyond the standard protocol.

### 2.5. VR Intervention

Patients allocated to the intervention group underwent OH as stated above, with the addition of a VR experience. VR environments were provided by a portable, standalone VR headset PICO G2 (Pico XR, Mountain View, CA, USA), with a head-mounted display with built-in audio speakers. Prior to the hysteroscopy procedure, patients in the VR group viewed a 7 min conscious and guided relaxation “body-scan” procedure, a recognized relaxation technique in mindfulness and meditation supported by scientific evidence [33] (environment developed by XRHealth (R)). Once the OH procedure began, a different scenario was displayed with patients immersed in a distracting 3D environment called “Under the Sea”, representing a videogame-like environment where patients were asked to look for specific sea life (environment developed by XRHealth (R)). Participants were required to keep the VR device in place but could remove it if they experienced discomfort or any adverse effects. All equipment underwent proper cleaning with wipes before and after each procedure.

### 2.6. Outcomes and Measurements

The primary outcome measures were patient-reported pain scores during the procedure and 10 min after completion, measured using a Visual Analogue Scale (VAS) ranging from 0 to 10, where 0 represented “Absence of pain” and 10 “The worst pain conceivable”, which is a validated scale that is easy to use and able to detect significant changes [34]. The baseline characteristics of the participants were compiled and included the following: age, pre or postmenopausal stage, pregnancy history, pre-hysteroscopy State-Trait Anxiety Inventory (STAI) state (STAI-S) and trait (STAIT-T) scores, cervical and endometrial preparation for the procedure, diagnosis and the protocol carried out in the patient.

Secondary outcomes included objective parameters related to pain and anxiety (heart rate, blood pressure, sweating) before, during, and after the hysteroscopic procedure. To collect these data, all participants underwent cardiac parameter monitoring before and after the procedure using a validated blood pressure monitor (OMRON M2 Plus, OMRON, Kyoto, Japan). Additionally, they were equipped with a Fitbit Charge 3 device (Fitbit Inc., San Francisco, CA, USA) to measure average heart rate and an Esense Skin Response device (Mindfield Biosystems, Gronau, Germany) to evaluate sweating through increases in skin conductance, since changes in these parameters have been defined as stress indicators [35,36,37,38].

Before the procedure, the participants’ anxiety status was evaluated using the validated STAI, a psychological tool divided into two questionnaires [39]. The STAI-S questionnaire defines the patient’s anxiety at a specific moment, describing the current emotional state. On the other hand, the STAIT-T questionnaire defines anxiety as a personality trait, describing the patient’s tendency to experience anxiety in diverse situations over time. Each questionnaire includes 20 statements, with participants indicating their level of agreement on a scale from 0 to 3, where 0 represents “Total disagreement” and 3 indicates “Total agreement”. The total sum of the items was calculated and ranged between 0 and 60, with relaxation-related items scoring in reverse. A threshold STAI score of 24 was established to classify patients with normal or high anxiety status/trait. This threshold corresponds to the average of Spanish women and also p50 of the STAI-T distribution [40].

After the process, all participants completed a final questionnaire assessing their experience with the hysteroscopic procedure and a separate questionnaire specifically related to using the VR device and the environments displayed to evaluate patient satisfaction, with this strategy as a potential alternative for reducing gynecological pain and discomfort associated with OH.

### 2.7. Statistical Analysis

Quantitative measures were summarized using the mean and standard deviation (SD), while qualitative measures were described using frequencies and percentages. The main analysis focused on comparing quantitative outcomes between study groups using an unpaired Student’s *t*-test. The *t*-test was applied under the assumption of independence of observations and heteroscedasticity; therefore, the variance was estimated using the Welch (or Satterthwaite) approximation. The analyses were performed using the compareGroups R package (v.4.8.0) [41]. The effect size was computed using Cohen’s d approach.

The same approach was performed by stratifying the data based on the individual characteristics of the participants to assess the impact of VR intervention in specific profiles. Stratification factors included baseline STAI-T score (under or over 24 points), menopausal status (premenopausal or postmenopausal), pregnancy history (parous or non-parous) and vaginal or non-vaginal delivery in childbirth. A complete case analysis was conducted, excluding missing values from each analysis by removing data from participants with incomplete information. A *p*-value of <0.05 was considered statistically significant, and 95% confidence intervals were applied without accounting for multiple testing corrections. Data management and statistical analyses were performed using the R software package (v.4.3.0) [42].

## 3. Results

### 3.1. Baseline Characteristics of the Study Groups

No baseline differences were observed between groups regarding clinical variables, anxiety or stress levels of the participants. Both groups also presented similar distributions in the diagnosis of the participants and the procedures performed (Table 1).

### 3.2. Effects of VR on Pain Perception and Stress

**Figure 2:**
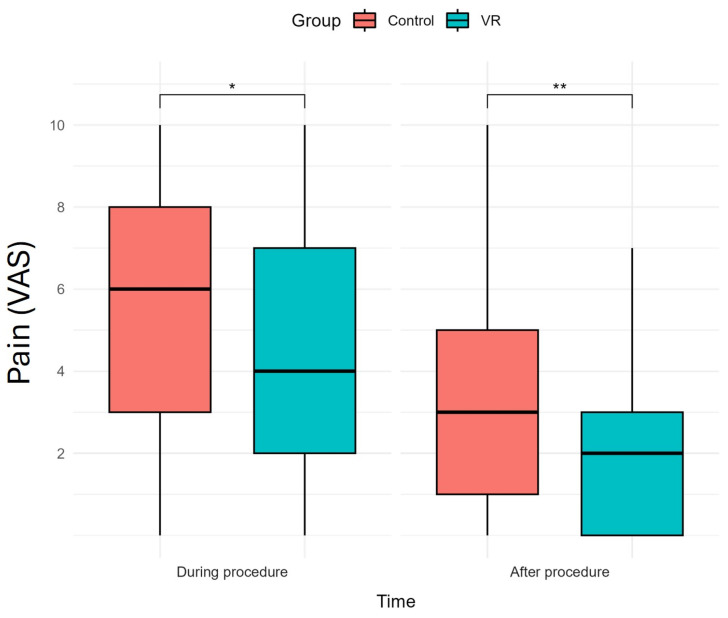
Boxplot of VR effects on perceived pain by patients in the control (orange) and VR group (green) during and after office hysteroscopy (*n* = 179). (* *p* < 0.05 ** *p* < 0.001). Note: V*R*, virtual reality; VAS, Visual Analogue Scale. The box represents the range in which the middle 50% of all values lie, with the lower end indicating the 1st quartile and the upper end the 3rd quartile.

Reported pain during and after the procedure is shown in Table 2 and Figure 2 (effect sizes described in Table S1). Patients in the VR group reported significantly lower pain levels compared to the control group during hysteroscopy (4.51 vs. 5.59 on the VAS, *p*-value = 0.013). These differences became even more pronounced 10 min post-procedure, with the VR group reporting significantly lower pain levels (2.09 vs. 3.33, *p*-value = 0.001).

Both groups exhibited comparable basal systolic and diastolic arterial pressure before hysteroscopy, which remained unaltered after the procedure without significant differences between the control and the VR groups (Table 2). Skin conductance measurements in the control group were comparable to those in the VR group, with VR not influencing this parameter. However, while the baseline heart rate (HR) was identical in both groups, a significant increase was observed in the VR group following the hysteroscopy (70.1 vs. 73.6, *p*-value = 0.027) (Table 2).

### 3.3. STAI-T Stratification

**Figure 3:**
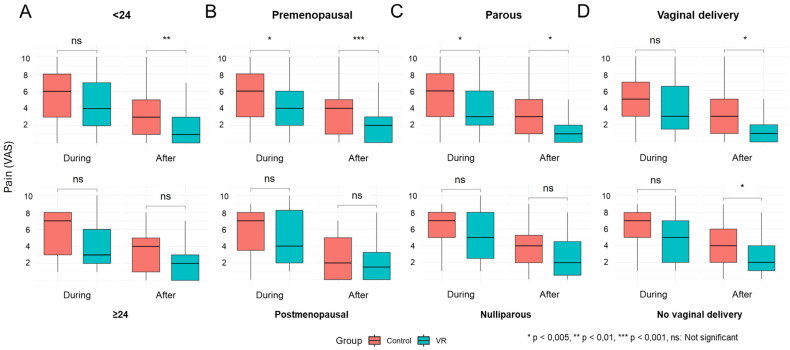
Boxplot of VR effects on perceived pain by patients in the control (orange) and VR group (green) during and after office hysteroscopy across stratified subgroups. (**A**) According to STAI-T score < 24 (up) or ≥24 (down). (**B**) According to the menopausal stage, premenopausal (up) or postmenopausal (down). (**C**) According to pregnancy history, parous (up) or nulliparous (down). (**D**) According to vaginal delivery, yes (up) or no (down). (* *p* < 0.05 ** *p* < 0.01 *** *p* < 0.001 ns: not significant.) Note: VR, virtual reality.

In total, 120 patients (63 from the control group and 57 from the VR group) scored below 24 points, while the remaining 38 (17 from the control and 21 from the VR groups) scored equal to or higher than 24. One patient did not complete the questionnaire properly and was excluded from this analysis. In the low-anxiety group, pain perception during the procedure was significantly lower in the VR group compared to the control group (5.56 vs. 4.56, *p*-value = 0.052), and these differences persisted 10 min post-intervention (3.29 vs. 1.95, *p*-value = 0.002) (Figure 3A, Table S2). Additionally, the final HR was higher in the VR group than in the control group (69.9 vs. 73.8, *p*-value = 0.047). No significant differences were observed between groups in arterial pressure or skin conductance. In contrast, participants with high anxiety scores showed no significant differences in pain perception or objective parameters between the control and VR groups, either during the procedure or 10 min after its completion.

### 3.4. Menopausal Stage Stratification

VR significantly reduced pain perception in premenopausal women (5.58 vs. 4.37, *p*-value = 0.012) but did not affect postmenopausal patients (Figure 3B, Table S3). These differences persisted in pain perceived after the procedure (3.63 vs. 2.05, *p*-value < 0.001), while these changes were not statistically significant in postmenopausal women. According to menopausal stratification, cardiac and skin conductance parameters remained unaffected.

### 3.5. Pregnancy History Stratification

A total of 100 patients had a history of pregnancy (parous), while 59 were non-parous (Figure 3C, Table S4). In parous women, VR significantly reduced pain during the procedure (5.29 vs. 4.08, *p*-value = 0.028). This effect was not observed in non-parous women. The differences persisted post-procedure, with parous women continuing to report lower pain perception levels when submitted to VR (3.04 vs. 1.79, *p*-value = 0.01), while no significant changes were observed in non-parous women. Additionally, VR significantly increased HR in parous women (70.2 vs. 74.8, *p*-value = 0.019). No differences were observed in other parameters of pregnancy history stratification.

### 3.6. Vaginal Delivery Stratification

Eighty-three women had a history of vaginal delivery, and seventy-six women had no pregnancy history or the delivery route was through cesarian section (no vaginal delivery). Unlike previous stratifications, no significant differences were observed in pain perception during the hysteroscopy between the control and VR groups, regardless of vaginal delivery history. However, a non-significant trend was observed in the no vaginal delivery subpopulation (6.08 vs. 4.94, *p*-value = 0.059) (Figure 3D, Table S5). Nevertheless, VR significantly reduced pain perception post-procedure in both groups (2.83 vs. 1.65, *p*-value = 0.027 for vaginal delivery and 3.83 vs. 2.61, *p*-value = 0.031 for non-vaginal delivery). The HR averages were comparable across both groups, and no significant differences were identified in other cardiac parameters or in skin conductance.

### 3.7. Patient Satisfaction Regarding the Hysteroscopic Procedure and the Use of VR

No difference regarding overall procedure satisfaction, intimacy level, quality of the information about the procedure or satisfaction with the duration of intervention was observed between groups (Table 3). Although not statistically significant, 27 patients in the control group (38.8%) and 16 in the intervention group (20,3%) experienced nausea or dizziness at some point during the procedure.

Patient feedback regarding satisfaction with the VR device and the generated 3D environment is listed in Table 4. Among VR users, most participants (72.2%) found the headset very comfortable, while 21.5% described it as relatively comfortable. Only four patients reported medium comfort, and one patient experienced relative uncomfortableness.

In evaluating the quality of the mindfulness 3D environment, 58.2% of patients rated it as very good and 38% as good, with two reports of regular quality and one case of bad quality. The assessment of the quality of the 3D environments followed a similar trend: 55.7% rated it as very good and 34.2% as good, with seven participants describing it as regular and one as bad.

Overall, the VR experience was highly satisfactory, with 55.7% of users indicating they would definitively use it again and 30.4% considering it likely for future use. Eight participants were undecided, while only three would not consider using it again.

Finally, 69.6% of the patients reported being very satisfied with the overall VR experience, while 25.3% were somewhat satisfied. Only two patients were slightly satisfied; one was somewhat dissatisfied, and another was very dissatisfied.

## 4. Discussion

The present study demonstrates that VR significantly reduces pain associated with OH, both during the procedure and 10 min post-procedure. Furthermore, our findings underscore the importance of individual patient factors. Baseline anxiety and clinical variables such as the menopausal state, the pregnancy history or vaginal delivery birth can significantly influence the effectiveness of this therapeutic approach.

Non-pharmacological interventions like music or hypnosis have recently been used in clinical settings to reduce perceived pain, though with mixed results [43,44,45]. Nevertheless, VR has emerged as an effective option for alleviating perceived pain during invasive medical procedures. The results of our study demonstrate that VR significantly reduces the pain perceived during and after OH, aligning with previous studies [28]. In a comparable work, Pelazas et al. also reported a decrease in pain levels in patients using VR, although anxiety was not assessed [8]. However, some other contradictory results have been published. Fouks et al. found no benefit of VR on pain during OH, though they emphasized that their procedures were more prolonged, which may account for elevated pain perception [29]. Furthermore, patients were asked about analgesic use, which may have introduced a selection bias. Another study by Sewel et al. reported contradictory outcomes, reporting no benefits of VR on pain perception [39]. This study also involved the operator’s discretionary use of extra local anesthetics and analgesics, which may have influenced the observed results. Fouks and Sewel’s studies also had fewer participants than ours, a limitation frequently described as crucial in this type of research.

In our work, we observed that the final HR significantly changed with the use of VR. While strategies like music have been shown to decrease HR due to their calming effects [44], VR has exhibited the opposite outcome [29]. This likely stems from the immersive nature of the 3D scenarios, which are often unfamiliar to patients and have a stimulating and excitatory impact that increases their HR [18]. This is consistent with previous findings that report that VR can provoke solid psychophysiological sensations [46,47].

Our findings indicate that the efficacy of VR in reducing pain is associated with some patients’ clinical characteristics, specifically their STAI-T score. The results showed that VR significantly reduced pain in patients with an STAI-T score below the cut-off of 24 points (the median for the female Spanish population of the STAI-T score) [40]. This result, although unexpected, supports the idea of VR functioning as a distraction tool. According to the control gate theory [48], pain perception is multimodal and influenced by additional stimuli; thus, high anxiety levels can impair an individual’s ability to focus on a distraction, influencing pain perception. Therefore, patients with higher anxiety scores may have more difficulty engaging with or paying attention to the 3D virtual environment, diminishing VR’s analgesic effect. High-anxiety individuals often experience intrusive thoughts and attentional interferences, which reduce their capacity to focus on VR as a pain management tool [49]. This suggests that while VR can be effective in patients with lower anxiety, its benefit might be reduced in those with high anxiety.

Secondly, our results showed that VR was associated with a significant decrease in pain reported by premenopausal women, but this significance was not reached in the postmenopausal group. Research indicates that postmenopausal women tend to report higher pain levels during hysteroscopy due to physiological changes associated with menopause, such as increased vaginal dryness and cervical stenosis, restricting hysteroscope access through the cervical canal and increasing pain levels [50]. Given this, it is unsurprising that postmenopausal women often require more anesthetics [51] and also respond better to local analgesics [17]. These physiological factors likely contribute to the moderate but effective pain relief observed with VR in premenopausal women, as this population may not fully benefit from anesthetics but remains susceptible to non-pharmacological interventions like VR due to their relatively lower overall pain. This makes VR a viable alternative for managing pain in premenopausal women, offering an analgesic effect when standard treatments may be insufficient.

A similar conclusion can be drawn from the pregnancy history stratification of our study. The results showed that the use of VR significantly reduced OH-associated pain in women with a pregnancy history. Nulliparous women are less likely to have cervical canal expansion, making the hysteroscopy process more painful [12,52,53,54]. Their pain levels may interfere with the efficacy of other approaches, but VR has proven effective in reducing pain in this group. Our findings, in line with the existing literature, highlight that women with a history of vaginal delivery generally experience less pain during hysteroscopy due to a naturally more dilated cervical canal. This postulates the pregnancy history of the patients as a crucial factor in determining VR effectiveness [50]. The results strengthen the hypothesis that VR is more effective in populations experiencing lower baseline hysteroscopy pain. In such cases, the subtle but noticeable analgesic effects of VR can be better observed. Nevertheless, additional studies should clarify the clinical relevance of the observed findings.

Our study incorporated skin conductance evaluations during the procedure to measure anxiety and stress in real time. This approach has been validated as a reliable marker of anxiety across different settings [35,36,37]. However, our results showed no significant differences in skin conductance or the other cardiac parameters measured throughout the procedure. These findings support the hypothesis that pain perception is more closely related to trait anxiety than to state anxiety, as suggested by Kokanali [16]. Previous research has indicated that technologies like VR may help reduce OH-associated anxiety [28,31,55]. However, our analysis is the first to demonstrate with objective parameters that anxiety levels do not fluctuate significantly during the procedure. Instead, it appears that baseline anxiety levels influence how pain is perceived both during and after the procedure, which might explain the conflicting outcomes in the literature regarding anxiety reduction.

This study represents the most comprehensive trial to date evaluating the efficacy of VR in reducing OH-associated pain. Previous research on the topic had smaller sample sizes [8,28,29,30,31], leading to inconsistent results and a call for larger trials involving more participants to obtain robust evidence for VR effectiveness [55,56,57].

Our research also addressed critical points raised by previous works. For instance, it has been proposed that different 3D environments could increase variability [55]. In our study, participants were exposed to the same 3D environment, reducing heterogeneity in the outcomes; however, Pelazas et al. suggested that more significant results could be obtained by selecting a specific 3D reality by the patient. It is hypothesized that adapted 3D experiences, where patients can choose a 3D immersion based on their preferences, can have more significant results. [8]. A comparable strategy was applied in music-based interventions, where different styles were tailored to individual tastes [44]. Our study groups also represented the general population, including patients undergoing a broad spectrum of gynecological procedures instead of focusing on one intervention, a key factor as exposed previously [31].

By stratifying the data according to clinical characteristics, we examined subpopulations more likely to benefit from VR and/or to confirm the consistency of the overall findings. Finally, analyzing anxiety during hysteroscopy remains a complex challenge due to the characteristics of the study. Nevertheless, we have evaluated anxiety levels in real-time for the first time during OH. Our study also adhered to recommendations by Malaris et al., which encouraged the authors to gather patient feedback on the 3D environment used in VR interventions [22]. The results from the final satisfaction questionnaires highlighted that the technique was widely regarded as comfortable and of high quality. Most participants expressed a willingness to use the technology again and showed a high likelihood of recommending it to others, reinforcing VR’s feasibility and patient approval for future application in medical settings.

### Limitations and Strengths of This Study

This study has certain limitations that should be considered when interpreting the findings. First, the characteristics of the study impede the conduct of a blind trial, which might result in the underreporting of pain by the VR group and overreporting by the control group. Secondly, while we aimed to recruit more participants, the COVID-19 pandemic severely restricted the recruitment. Additionally, this protocol did not incorporate headphones for sound stimulation, which some authors suggest enhances the immersive and distracting qualities of the 3D environment. However, other authors consider that complete isolation can have detrimental effects on pain perception and anxiety levels [31]. Another limitation was the variability in the hysteroscopic equipment used, as different instruments were adapted to patients’ needs. Moreover, including various gynecological procedures could contribute to the heterogeneity of results. Another concern is the possible recall bias from patients completing the VAS questionnaire post-procedure. Fouks et al. also highlighted the potential placebo effect of VR on post-procedure pain, suggesting that further analysis is needed to clarify the full impact of VR [29]. Finally, the results obtained from the stratification should be cautiously considered since the separation into unbalanced groups of patients might affect the statistical power of the study.

Despite these limitations, the study has several strengths that contribute to its validity and relevance in clinical settings. First, the use of real-time anxiety measurement through skin conductance represents a novel and objective method for evaluating anxiety during medical procedures. These objective data enhance the reliability of our findings compared to studies relying solely on subjective self-report measures.

Additionally, our large and diverse sample, including patients with a range of gynecological conditions, provides a more comprehensive view of VR’s effectiveness in different clinical scenarios. By stratifying the data based on key clinical factors, such as anxiety levels, menopausal status, and pregnancy history, our study offers valuable insights into how individual patient characteristics could influence the effectiveness of VR interventions. These data may help identify specific patient subgroups that may benefit most from VR and individualize the analgesic strategy for OH procedures.

Furthermore, the high patient satisfaction with the VR experience, as indicated by the post-procedure surveys, supports the feasibility and acceptability of VR as a non-pharmacological pain management tool in medical procedures.

## 5. Conclusions

Virtual reality effectively reduces pain associated with OH, and its effectiveness depends on patient-specific variables. The anxiety trait and the gynecological clinical history, as well as the menopausal state, condition the efficacy of VR to decrease the pain associated with hysteroscopy. Further development of VR devices and the environments displayed may be an effective strategy for pain management that is affordable for medical settings.

## Figures and Tables

**Table 1:** Baseline characteristics of the study population.

Variable	CTL (*n* = 80)	VR (*n* = 79)
Age, mean years (SD)	45.6 (9.9)	43.3 (10)
Menopause, *n* (%):		
Premenopause	57 (71.20)	63 (79.70)
Postmenopause	23 (28.70)	16 (20.30)
Pregnancy history, *n* (%):		
Nulliparous	32 (40)	27 (34.20)
Parous	48 (60)	52 (65.80)
Initial STAI-T Score, mean (SD)	16.50 (8.65)	18.10 (7.34)
Initial STAI-S Score, mean (SD)	18.30 (9.69)	19.20 (9.85)
Cervical preparation, *n* (%):		
No	72 (90)	72 (91.10)
Yes	8 (10)	7 (8.86)
Endometrial preparation, *n* (%):		
No	73 (91.20)	66 (83.50)
Yes	7 (8.75)	13 (16.50)
Diagnosis, *n* (%):		
Normality	10 (12.50)	13 (16.50)
Endometrial Polyp	44 (55)	39 (49.40)
Fibroid	9 (11.20)	8 (10.10)
Retention of IUD	7 (8.75)	9 (11.40)
RPOC	3 (3.75)	4 (5.06)
Intrauterine adhesions	2 (2.50)	2 (2.53)
Others	5 (6.25)	4 (5.06)
Procedure, *n* (%):		
None	10 (12.50)	14 (17.70)
Targeted Biopsy	14 (17.50)	12 (15.20)
Polypectomy	45 (56.20)	39 (49.40)
RPOC removal	3 (3.75)	4 (5.06)
IUD removal	7 (8.75)	8 (10.10)
Oppium technique	1 (1.25)	2 (2.53)

Note: CTL, control; VR, virtual reality; STAI, State-Trait Anxiety Inventory; IUD, Intrauterine Device; RPOC, Retained products of conception; SD, standard deviation.

**Table 2:** Comparison of pain- and stress-related variables.

Variable	Control (*n* = 80)	VR (*n* = 79)	*p*-Value	Mean Diff (CI)
Pain intra, mean VAS (SD)	5.59 (2.50)	4.51 (2.90)	0.013	−1.08 (−1.93–−0.23)
Pain post, mean VAS (SD)	3.33 (2.57)	2.09 (2.24)	0.001	−1.24 (−1.99–−0.48)
Basal Heart Rate, mean bpm (SD)	74.70 (8.17)	76.60 (9.69)	0.170	1.97 (−0.86–4.81)
Final Heart Rate, mean bpm (SD)	70.10 (9.41)	73.60 (10.50)	0.027	−3.56 (0.41–6.71)
Basal Systolic Blood Pressure, mean mmHg (SD)	125 (17.90)	124 (16.20)	0.641	−1.27 (−6.65–4.10)
Final Systolic Blood Pressure, mean mmHg (SD)	119 (17.90)	120 (15.60)	0.759	0.82 (−4.47–6.12)
Basal Diastolic Blood Pressure, mean mmHg (SD)	79.10 (12.40)	78 (11.10)	0.572	−1.06 (−4.77–2.64)
Final Diastolic Blood Pressure, mean mmHg (SD)	79.20 (10.70)	79.40 (12.30)	0.951	0.11 (−3.52–3.75)
Maximum Skin Conductance, mean µS (SD)	2485 (2667)	2264 (1866)	0.562	−221.24 (−973.44–530.966)
Increase in Skin Conductance, mean µS (SD)	1367 (1921)	1070 (1139)	0.257	−297.16 (−813.79–219.48)

Note: CTL, control; CI, confidence interval; bpm, beats per minute; Mean diff, mean difference; VR, virtual reality; VAS, Visual Analogue Scale; SD, standard deviation.

**Table 3:** Patient’s satisfaction with the procedure.

Variable	CTL (*n* = 80)	VR (*n* = 79)
Overall satisfaction with the procedure, *n* (%)		
Very satisfied	71 (88.80)	73 (92.40)
Somewhat satisfied	7 (8.80)	5 (6.30)
Little satisfied	0 (0)	0 (0)
Somewhat dissatisfied	1 (1.30)	0 (0)
Very dissatisfied	1 (1.30)	1 (1.30)
Assessment of nurse’s attendance, *n* (%)		
Very kind	77 (96.30)	77 (97.50)
Relatively kind	3 (3.80)	2 (2.50)
Neutral	0 (0)	0 (0)
Relatively unkind	0 (0)	0 (0)
Very unkind	0 (0)	0 (0)
Assessment of gynecologist’s attendance, *n* (%)		
Very kind	79 (98.80)	76 (96.20)
Relatively kind	1 (1.30)	3 (3.80)
Neutral	0 (0)	0 (0)
Relatively unkind	0 (0)	0 (0)
Very unkind	0 (0)	0 (0)
Level of intimacy during the procedure, *n* (%)		
High	67 (83.80)	62 (78.50)
Good	12 (15)	16 (20.3)
Moderate	1 (1.30)	1 (1.30)
Scarce	0 (0)	0 (0)
Bad	0 (0)	0 (0)
Quality level of the information received, *n* (%)		
High	73 (91.30)	75 (94.90)
Good	5 (6.30)	4 (5.10)
Moderate	1 (1.30)	0 (0)
Scarce	1 (1.30)	0 (0)
Bad	0 (0)	0 (0)
Duration of the procedure, *n* (%)		
Very acceptable	68 (85)	62 (78.50)
Slightly acceptable	3 (3.80)	6 (7.60)
Correct	6 (7.50)	11 (13.90)
Slightly prolonged	2 (2.50)	0 (0)
Very prolonged	1 (1.30)	0 (0)
Presence of nausea or dizziness, *n* (%)		
No	53 (66.30)	63 (79.90)
In certain moments	15 (18.80)	10 (12.70)
Slightly	8 (10)	6 (7.60)
Quite a few	4 (5)	0 (0)
During all the procedure	0 (0)	0 (0)

Note: CTL, control; VR, virtual reality.

**Table 4:** Patients’ satisfaction regarding the use of VR devices and environments.

Variable	Overall (*n* = 79)
VR headset comfort, *n* (%)	
Very comfortable	57 (72.20)
Relatively comfortable	17 (21.50)
Somewhat comfortable	4 (5.10)
Relatively uncomfortable	1 (1.30)
Very uncomfortable	0 (0)
Quality of the mindfulness 3D environment, *n* (%)	
Very good	46 (58.20)
Good	30 (38)
Regular	2 (2.50)
Bad	1 (1.30)
Very bad	0 (0)
Quality of the 3D environment during the procedure, *n* (%)	
Very good	44 (55.70)
Good	27 (34.20)
Regular	7 (8.90)
Bad	1 (1.30)
Very bad	0 (0)
To what extent would use VR again in other treatments, *n* (%)	
Not for sure	3 (3.80)
Probably not	0 (0)
Maybe	8 (10.10)
Probably yes	24 (30.40)
Yes for sure	44 (55.70)
Overall satisfaction with VR experience, *n* (%)	
Very satisfied	55 (69.60)
Somewhat satisfied	20 (25.30)
Little satisfied	2 (2.50)
Somewhat dissatisfied	1 (1.30)
Very dissatisfied	1 (1.30)
Overall satisfaction with VR procedure, *n* (%)	
Very nice	55 (69.60)
Quite nice	20 (25.30)
Poor	3 (3.80)
Unpleasant	1 (1.30)
Very bad	0 (0)

Note: CTL, control; VR, virtual reality; 3D, three-dimensional.

## Data Availability

The datasets presented in this article are not readily available because they belong to medical records.

## Supplementary Materials

The following supporting material can be downloaded at: https://www.mdpi.com/article/10.3390/healthcare13020131/s1, Table S1. Effect sizes (Cohen’s d) for each outcome measure of pain- and stress-related variables; Table S2. Comparative of pain and stress variables according to stratification based on STAI-T results; Table S3. Comparative of pain and stress variables according to stratification based on menopausal stage, Table S4. Comparative of pain and stress variables according to stratification based on pregnancy history; Table S5. Comparative of pain and stress variables according to stratification based on history of vaginal delivery.

## Abbreviations

The following abbreviations are used in this manuscript:

OH	office hysteroscopy
STAI	State-Trait Anxiety Inventory
RPOC	retained products of conception
IUD	Intrauterine Device
NSAIDs	nonsteroidal anti-inflammatory drugs
VAS	Visual Analogue Scale
HR	heart rate
VR	virtual reality
3D	three-dimensional
