# Supplementary material for: Effectiveness of Virtual Reality in Reducing Pain and Stress During Office Hysteroscopy: A Randomized Controlled Trial
Source: Healthcare (Basel). 2025 Jan 12;13(2):131. doi: 10.3390/healthcare13020131 (PMC11765363; doi:10.3390/healthcare13020131)
Supplement: Supplementary file 1 [file healthcare-13-00131-s001.zip › Supplementary Table S1.pdf]

Variable	Cohen's d	CI
**Pain intra,** mean VAS (SD)	-0.40	-0.72—-0.08
**Pain post,** mean VAS (SD)	-0.51	-0.83—-0.20
**Basal Heart Rate,** mean bpm (SD)	0.22	-0.10—0.54
**Final Heart Rate,** mean bpm (SD)	0.36	0.04—0.68
**Basal Systolic Blood Pressure,** mean mmHg (SD)	-0.07	-0.39—0.24
**Final Systolic Blood Pressure,** mean mmHg (SD)	0.05	-0.27—0.36
**Basal Diastolic Blood Pressure,** mean mmHg (SD)	-0.09	-0.41—0.23
**Final Diastolic Blood Pressure,** mean mmHg (SD)	0.01	-0.31—0.33
**Maximum Skin Conductance,** mean μS (SD)	-0.10	-0.42—0.23
**Increase in Skin Conductance,** mean μS (SD)	-0.19	-0.52—0.14

Note: CTL, Control; CI, confidence interval; bpm, beats per minute; Mean diff, mean difference; VR, Virtual Reality; VAS, Visual Analogue Scale; SD, Standard Deviation
