# Supplementary material for: Effectiveness of Virtual Reality in Reducing Pain and Stress During Office Hysteroscopy: A Randomized Controlled Trial
Source: Healthcare (Basel). 2025 Jan 12;13(2):131. doi: 10.3390/healthcare13020131 (PMC11765363; doi:10.3390/healthcare13020131)
Supplement: Supplementary file 1 [file healthcare-13-00131-s001.zip › Supplementary Table S2.pdf]

| | STAI Score | | | | | | | |
|---|---|---|---|---|---|---|---|---|
| | **≥24** | | | | **<24** | | | |
| **Variable** | **CTL** (n=17) | **VR** (n=21) | **p-value** | **Mean diff (CI)** | **CTL** (n=63) | **VR** (n=57) | **p-value** | **Mean diff (CI)** |
| **Pain intra,** mean VAS (SD) | 5.71 (2.42) | 4.48 (2.80) | 0.155 | -1.23 (-2.95—0.49) | 5.56 (2.54) | 4.56 (2.96) | **0.052** | -0.99 (-2.00—0.01) |
| **Pain post,** mean VAS (SD) | 3.47 (2.58) | 2.48 (2.75) | 0.259 | -0.99 (-2.75—0.76) | 3.29 (2.59) | 1.95 (2.05) | **0.002** | -1.34 (-2.18—-0.50) |
| **Basal Heart Rate,** mean bpm (SD) | 73.60 (7.74) | 76.80 (8.91) | 0.241 | 3.22 (-2.26—8.70) | 75 (8.32) | 76.20 (9.78) | 0.453 | 1.27 (-2.07—4.61) |
| **Final Heart Rate,** mean bpm (SD) | 70.80 (8.05) | 72 (6.26) | 0.594 | 1.28 (-3.58—6.15) | 69.90 (9.79) | 73.80 (11.40) | **0.047** | 3.96 (0.05—7.88) |
| **Basal Systolic Blood Pressure,** mean mmHg (SD) | 125 (19.50) | 122 (16) | 0.543 | -3.62 (-15.59—8.36) | 125 (17.70) | 124 (16.10) | 0.782 | -0.86 (-7.01—5.29) |
| **Final Systolic Blood Pressure,** mean mmHg (SD) | 121 (20.10) | 119 (9.84) | 0.644 | -2.50 (-13.53—8.53) | 119 (17.40) | 120 (17.40) | 0.642 | 1.50 (-4.86—7.85) |
| **Basal Diastolic Blood Pressure,** mean mmHg (SD) | 83.40 (14.20) | 78.90 (10.30) | 0.283 | -4.51 (-12.94—3.93) | 77.90 (11.60) | 77.30 (11.20) | 0.771 | -0.61 (-4.79—3.56) |
| **Final Diastolic Blood Pressure,** mean mmHg (SD) | 81.80 (12.10) | 78.50 (11.40) | 0.406 | -3.24 (-11.06—4.58) | 78.60 (10.20) | 79.40 (12.70) | 0.699 | 0.829 (-3.41—5.07) |
| **Maximum Skin Conductance,** mean µS (SD) | 2966 (3633) | 2101 (1040) | 0.384 | -864.60 (-2916.30—1187.10) | 2361 (2381) | 2359 (2101) | 0.996 | -1.88 (-845.16—841.41) |
| **Increase in Skin Conductance,** mean µS (SD) | 2070 (3158) | 1067 (701) | 0.246 | -1002.86 (-2772.30—766.60) | 1186 (1431) | 1091 (1273) | 0.712 | -94.98 (-603.58—413.63) |

Note: *CTL*, Control; *CI*, confidence interval; *bpm*, beats per minute; *Mean diff*, mean difference; *VR*, Virtual Reality; *VAS*, Visual Analogue Scale; *SD*, Standard Deviation
