# Supplementary material for: Effectiveness of Virtual Reality in Reducing Pain and Stress During Office Hysteroscopy: A Randomized Controlled Trial
Source: Healthcare (Basel). 2025 Jan 12;13(2):131. doi: 10.3390/healthcare13020131 (PMC11765363; doi:10.3390/healthcare13020131)
Supplement: Supplementary file 1 [file healthcare-13-00131-s001.zip › Supplementary Table S3.pdf]

	Menopausal Stage							
	Premenopause				Premenopause			
**Variable**	**CTL** (n=57)	**VR** (n=63)	**p-value**	**Mean diff (CI)**	**CTL** (n=23)	**VR** (n=16)	**p-value**	**Mean diff (CI)**
**Pain intra,** mean VAS (SD)	5.58 (2.47)	4.37 (2.77)	**0.012**	-1.21 (-2.16—-0.27)	5.61 (2.64)	5.06 (3.42)	0.595	-0.55 (-2.63—1.54)
**Pain post,** mean VAS (SD)	3.63 (2.57)	2.05 (2.16)	**<0.001**	-1.58 (-2.45—-0.72)	2.57 (2.45)	2.25 (2.59)	0.705	-0.32 (-2.00—1.37)
**Basal Heart Rate,** mean bpm (SD)	75.10 (8.12)	76.50 (10.0)	0.392	1.43 (-1.87—4.73)	73.70 (8.39)	77.30 (8.40)	0.207	3.59 (-2.10—9.29)
**Final Heart Rate,** mean bpm (SD)	70.10 (9.40)	73.70 (11.3)	0.062	3.59 (-0.18—7.36)	70 (9.63)	73.40 (6.09)	0.191	3.40 (-1.78—8.58)
**Basal Systolic Blood Pressure,** mean mmHg (SD)	119 (14.90)	121 (14.50)	0.594	1.44 (-3.90—6.78)	140 (16.7)	137 (16.50)	0.622	-2.74 (-13.99—8.50)
**Final Systolic Blood Pressure,** mean mmHg (SD)	113 (15.10)	116 (13.40)	0.209	3.31 (-1.88—8.51)	134 (15.7)	135 (16.30)	0.928	0.49 (-10.43—11.41)
**Basal Diastolic Blood Pressure,** mean mmHg (SD)	75.70 (11.20)	76.60 (10.5)	0.660	3.88 (-3.08—4.84)	87.50 (11.1)	84.0 (12.10)	0.378	-3.48 (-11.43—4.47)
**Final Diastolic Blood Pressure,** mean mmHg (SD)	77.20 (10.3)	77.30 (11.3)	0.974	0.06 (-3.87—3.99)	84.30 (10.1)	88 (12.80)	0.354	3.97 (-4.37—11.76)
**Maximum Skin Conductance,** mean µS (SD)	2515 (2716)	2275 (1992)	0.600	-239.06 (-1141.19—663.08)	2408 (2600)	2220 (1311)	0.781	-188.56 (-1563.44—1186.34)
**Increase in Skin Conductance,** mean µS (SD)	1456 (2121)	1071 (1219)	0.250	-384.53 (-1044.59—275.53)	1134 (1261)	1067 (774)	0.848	-66.68 (-770.98—637.61)

Note: *CTL*, Control; *CI*, confidence interval; *bpm*, beats per minute; *Mean diff*, mean difference; *VR*, Virtual Reality; *VAS*, Visual Analogue Scale; *SD*, Standard Deviation
