# Supplementary material for: Effectiveness of Virtual Reality in Reducing Pain and Stress During Office Hysteroscopy: A Randomized Controlled Trial
Source: Healthcare (Basel). 2025 Jan 12;13(2):131. doi: 10.3390/healthcare13020131 (PMC11765363; doi:10.3390/healthcare13020131)
Supplement: Supplementary file 1 [file healthcare-13-00131-s001.zip › Supplementary Table S4.pdf]

| | Pregnancy History | | | | | | | |
|---|---|---|---|---|---|---|---|---|
| | Parous | | | | Nulliparous | | | |
| **Variable** | **CTL** (n=48) | **VR** (n=52) | **p-value** | **Mean diff (CI)** | **CTL** (n=32) | **VR** (n=27) | **p-value** | **Mean diff (CI)** |
| **Pain intra,** mean VAS (SD) | 5.29 (2.56) | 4.08 (2.87) | **0.028** | -1.22 (-2.29—-0.14) | 6.03 (2.39) | 5.33 (2.83) | 0.316 | -0.70 (-2.08—0.69) |
| **Pain post,** mean VAS (SD) | 3.04 (2.65) | 1.79 (2.08) | **0.010** | -1.25 (-2.21—-0.30) | 3.75 (2.42) | 2.67 (2.45) | 0.095 | -1.08 (-2.36—0.19) |
| **Basal Heart Rate,** mean bpm (SD) | 75.40 (7.23) | 77.80 (9.03) | 0.136 | 2.45 (-0.79—5.69) | 73.60 (9.42) | 74.20 (10.70) | 0.835 | 0.57 (-4.88—6.02) |
| **Final Heart Rate,** mean bpm (SD) | 70.20 (7.77) | 74.80 (11.10) | **0.019** | 4.54 (0.76—8.32) | 69.80 (11.60) | 71.20 (8.81) | 0.601 | 1.42 (-3.99—6.83) |
| **Basal Systolic Blood Pressure,** mean mmHg (SD) | 126 (19.80) | 125 (16.40) | 0.866 | -0.62 (-7.87—6.64) | 124 (14.90) | 120 (15.30) | 0.429 | -3.22 (-11.32—4.88) |
| **Final Systolic Blood Pressure,** mean mmHg (SD) | 120 (17.30) | 122 (16.30) | 0.593 | 1.81 (-4.87—8.48) | 118 (19) | 116 (13.70) | 0.691 | -1.73 (-10.43—6.97) |
| **Basal Diastolic Blood Pressure,** mean mmHg (SD) | 80.30 (13.30) | 78.60 (11.40) | 0.492 | -1.72 (-6.66—3.22) | 77.20 (10.80) | 76.80 (10.60) | 0.884 | -0.42 (-6.13—5.30) |
| **Final Diastolic Blood Pressure,** mean mmHg (SD) | 79.30 (10) | 79.60 (12.30) | 0.913 | 0.25 (-4.20—4.69) | 79.20 (11.70) | 79 (12.50) | 0.952 | -0.20 (-6.74—6.34) |
| **Maximum Skin Conductance,** mean µS (SD) | 2170 (2259) | 2163 (1506) | 0.986 | -7.10 (-816.72—802.58) | 2937 (3149) | 2462 (2449) | 0.532 | -475.10 (-1990.25—1040.05) |
| **Increase in Skin Conductance,** mean µS (SD) | 1154 (1459) | 1047 (999) | 0.688 | -106.41 (-633.18—420.36) | 1674 (2431) | 1116 (1395) | 0.292 | -558.33 (-1612.67—496.02) |

Note: *CTL*, Control; *CI*, confidence interval; *bpm*, beats per minute; *Mean diff*, mean difference; *VR*, Virtual Reality; *VAS*, Visual Analogue Scale; *SD*, Standard Deviation
