# Supplementary material for: Effectiveness of Virtual Reality in Reducing Pain and Stress During Office Hysteroscopy: A Randomized Controlled Trial
Source: Healthcare (Basel). 2025 Jan 12;13(2):131. doi: 10.3390/healthcare13020131 (PMC11765363; doi:10.3390/healthcare13020131)
Supplement: Supplementary file 1 [file healthcare-13-00131-s001.zip › Supplementary Table S5.pdf]

	Vaginal Delivery History							
	Vaginal Delivery				No Vaginal Delivery			
**Variable**	**CTL** (n=40)	**VR** (n=43)	**p-value**	**Mean diff (CI)**	**CTL** (n=40)	**VR** (n=36)	**p-value**	**Mean diff (CI)**
**Pain intra,** mean VAS (SD)	5.10 (2.57)	4.14 (3.01)	0.121	-0.96 (-2.18—0.26)	6.08 (2.37)	4.94 (2.74)	0.059	-1.13 (-2.31—0.05)
**Pain post,** mean VAS (SD)	2.83 (2.60)	1.65 (2.08)	**0.027**	-1.17 (-2.21—-0.14)	3.83 (2.47)	2.61 (2.33)	**0.031**	-1.21 (-2.31—-0.15)
**Basal Heart Rate,** mean bpm (SD)	76 (6.95)	77.30 (9.34)	0.487	1.26 (-2.33—4.84)	73.30 (9.12)	75.90 (10.20)	0.268	2.53 (-1.99—7.06)
**Final Heart Rate,** mean bpm (SD)	70.40 (7.93)	74.30 (11.80)	0.081	3.88 (-0.48—8.24)	69.70 (10.80)	72.80 (8.71)	0.181	3.07 (-1.45—7.58)
**Basal Systolic Blood Pressure,** mean mmHg (SD)	127 (20.30)	125 (16.9)	0.612	-2.09 (-10.29—6.10)	123 (15.30)	123 (15.40)	0.851	-0.67 (-7.80—6.46)
**Final Systolic Blood Pressure,** mean mmHg (SD)	120 (16.40)	120 (16.60)	0.801	0.92 (-6.29—8.13)	119 (19.50)	119 (14.60)	0.879	0.61 (-7.31—8.52)
**Basal Diastolic Blood Pressure,** mean mmHg (SD)	80.80 (13.70)	77.80 (11.0)	0.279	-2.99 (-8.44—2.46)	77.30 (10.80)	78.30 (11.50)	0.726	0.92 (-4.28—6.11)
**Final Diastolic Blood Pressure,** mean mmHg (SD)	79 (9.94)	78.9 (12.80)	0.971	-0.09 (-5.08—4.89)	79.50 (11.50)	80 (11.80)	0.870	0.45 (-4.98—5.88)
**Maximum Skin Conductance,** mean µS (SD)	2224 (2384)	2206 (1463)	0.969	-17.75 (-935.70—900.21)	2740 (2927)	2336 (2294)	0.521	-403.65 (-1651.81—844.51)
**Increase in Skin Conductance,** mean µS (SD)	1197 (1514)	1065 (999)	0.657	-132.29 (-725.95—491.37)	1534 (2256)	1078 (1307)	0.299	-456.00 (-1326.86—414.87)

Note: *CTL*, Control; *CI*, confidence interval; *bpm*, beats per minute; *Mean diff*, mean difference; *VR*, Virtual Reality; *VAS*, Visual Analogue Scale; *SD*, Standard Deviation
